# Novel indices representing heterogeneous distributions of myocardial perfusion imaging

**DOI:** 10.1007/s12149-024-01920-w

**Published:** 2024-03-19

**Authors:** Misato Chimura, Tomohito Ohtani, Fusako Sera, Rie Higuchi, Kenji Kajitani, Kenichi Nakajima, Yasushi Sakata

**Affiliations:** 1 Department of Cardiovascular Medicine, Osaka University Graduate School of Medicine, 2-2 Yamadaoka, Suita, 565-0871 Japan; 2 Department of Nuclear Medicine/Functional Imaging and Artificial Intelligence, Kanazawa University Graduate School of Medicine, Kanazawa, Japan

**Keywords:** Myocardial heterogeneity, Myocardial perfusion imaging, Standard deviation, 95% bandwidth, Entropy

## Abstract

**Introduction:**

Heterogeneous distribution in myocardial perfusion images (MPI) obtained by scintigraphy is often observed in cardiac diseases with normal myocardial perfusion. However, quantitative assessments of such heterogeneity have not been established. We hypothesized that the heterogeneity in MPI can be quantitatively evaluated through histogram analysis, calculating the standard deviation (SD), the 95% bandwidth (BW95%), and entropy.

**Methods:**

We examined resting ^99m^Tc-MIBI images in 20 healthy subjects and 29 patients with cardiac disease who had none or very-mild reduced myocardial perfusion evaluated as a low summed rest score (0 to 4, the range of the studied healthy subjects). Two nuclear medicine specialists blindly divided them into two groups: non-heterogeneity or heterogeneity group, based solely on their visual assessments of heterogeneity on splash and polar maps generated from single-photon emission computed tomography (SPECT) images. The %uptake was determined by dividing the tracer count of each pixel by the tracer count of the pixel with the highest value in the LV myocardium. SD, BW95%, and entropy from histogram patterns were analyzed from the polar map data array of each %uptake. We investigated whether heterogeneity could be assessed using SD, BW95, and entropy in two groups classified by visual assessments. Additionally, we evaluated the area under the curve (AUC) to identify heterogeneity in the receiver operating characteristic curve analysis.

**Results:**

Based solely on visual assessments, 11 (22%) and 38 (78%) cases were classified into the non-heterogeneity and heterogeneity groups, respectively. The non-heterogeneity group consisted of only healthy subjects, and all patients with cardiac disease were classified into the heterogeneity group. The cases in the heterogeneity group had significantly higher values of heterogeneity indices (SD, BW95%, and entropy) in %uptake than those in the non-heterogeneity group (*p* < 0.05 for all). The AUCs of the heterogeneity indices were sufficiently high (AUCs > 0.90 for all) in distinguishing cases with visually heterogeneous distribution or patients with cardiac disease.

**Conclusions:**

Heterogeneity in MPI can be evaluated using SD, BW95%, and entropy through histogram analysis. These novel indices may help identify patients with subtle myocardial changes, even in images that show preserved perfusion (345/350).

## Introduction

The various heterogeneous distributions in myocardial perfusion images (MPI) of ^99m^Tc-methoxyisobutylisonitrile (MIBI) are observed in patients with cardiac diseases. For relatively prominent and localized myocardial perfusion abnormalities, it is possible to express the extent of myocardial damage using the summed rest score (SRS) of quantitative perfusion single-photon emission computed tomography (QPS) [1, 2]. However, even in cases with an SRS of 0–4, judged as normal or very-mild perfusion abnormality, heterogeneous perfusion might be visually observed, and no index to evaluate such subtle heterogeneous abnormality has been established. In this context, we hypothesized that distinguishing between regional perfusion distributions, whether they are homogeneous or heterogeneous, is possible through the analysis of histogram patterns using parameters like standard deviation (SD), bandwidth, and entropy. The level of heterogeneity can be quantified, with a more varied distribution resulting in a broader histogram pattern, whereas a uniform distribution would produce a narrower histogram.

Histogram analysis of the regional distribution has been used in phase analysis, in which Fourier transform the wall thickening of ECG-gated myocardial SPECT is used to assess the timing of contraction. Using polar and array maps of pixel-by-pixel phase values, a histogram analysis was conducted with a bin size of 0.05 (maximum 1.0). Parameters, such as SD, bandwidth at 95% (BW95%), and entropy of the phase distribution, were calculated [3, 4]. SD, BW95%, and entropy indicate the degree of variation in the phase, with larger values suggesting greater phase variation or disorder, and smaller values suggesting less phase variation or a more uniform distribution [5–8].

This study aimed to assess the quantification of the heterogeneous distribution on MPI by histogram analysis in cases with none or very-mild perfusion abnormality evaluated by SRS.

## Methods

### Study subjects

To analyze the cases with none or very-mild perfusion abnormality evaluated by SRS, we collected the data from healthy subjects and patients with cardiac diseases. From the volunteers recruited for this study, 20 healthy subjects (10 men and 10 women) ranging in age from their 30–70 s were prospectively enrolled after screening tests. The selection criteria for healthy subjects were as follows: no electrocardiographic abnormalities indicative of ischemia or arrhythmia, no underlying cardiac diseases, and normal blood pressure. Subjects with hypertension, dyslipidemia, or diabetes mellitus were also excluded, as were those who had inappropriate observations on echocardiography or abnormal laboratory data, and those who were obese with a BMI > 25 kg/m^2^. As SRS values of enrolled healthy subjects were 0–4, we retrospectively collected the data from 29 consecutive patients diagnosed with cardiac disease (9 dilated cardiomyopathy, 4 tachycardia-induced cardiomyopathy, 4 drug-induced cardiomyopathy, 2 valvular disease, 2 chronic myocarditis, 2 hypertensive heart disease, 1 hypertrophic cardiomyopathy, and 5 others), with an SRS of 0 to 4 on QPS, who visited Osaka University Hospital as outpatients or were hospitalized from March 2022 to August 2023. Baseline data, including echocardiographic information and body mass index (BMI), were collected on the day closest to the ^99m^Tc-MIBI scintigraphy. Transthoracic echocardiographic indices were obtained using standard methods as previously described [9]. This study was conducted in accordance with the Declaration of Helsinki and Good Clinical Practices and was approved by the Institutional Review Board of Osaka University Hospital (No. 21345-2).

### ^99m^Tc-MIBI scintigraphy protocol

A dose of 740 MBq of ^99m^Tc-MIBI was administered intravenously under a resting condition. SPECT imaging was obtained using a dual-head rotating camera (Symbia Intevo6, Erlangen, Germany), equipped with a low-energy high-resolution collimator. The rest SPECT images were obtained 30 min after administering the tracer. Projection images were obtained over 180° arc with an acquisition time of 40 s per image on a 64 × 64 matrix size with a 1.85 acquisition zoom. Energy window was centered at 140 keV with a 20% symmetric window. Non-gated SPECT data processed by ordered subset expectation maximization method (iteration, 3; subsets, 16; cutoff, 0.55 cycle/cm; and order, 8) was used without attenuation or scatter corrections.

### Assessments of MPI

A semi-quantitative analysis of regional tracer uptake in the LV myocardium was performed using a QPS protocol in rest images. The rest segment scores were automatically classified using a five-point scoring system: 0 for normal, 1 for mild defect, 2 for moderate defect, 3 for severe defect, and 4 for absent tracer uptake. This classification was applied to all 17 segments. To evaluate the overall assessment of the LV, the SRS was calculated by adding up the segment rest scores for all 17 segments [10, 11]. The %uptake was determined by dividing the tracer count of each pixel by the tracer count of the pixel with the highest value in the LV myocardium.

**Fig. 1:**
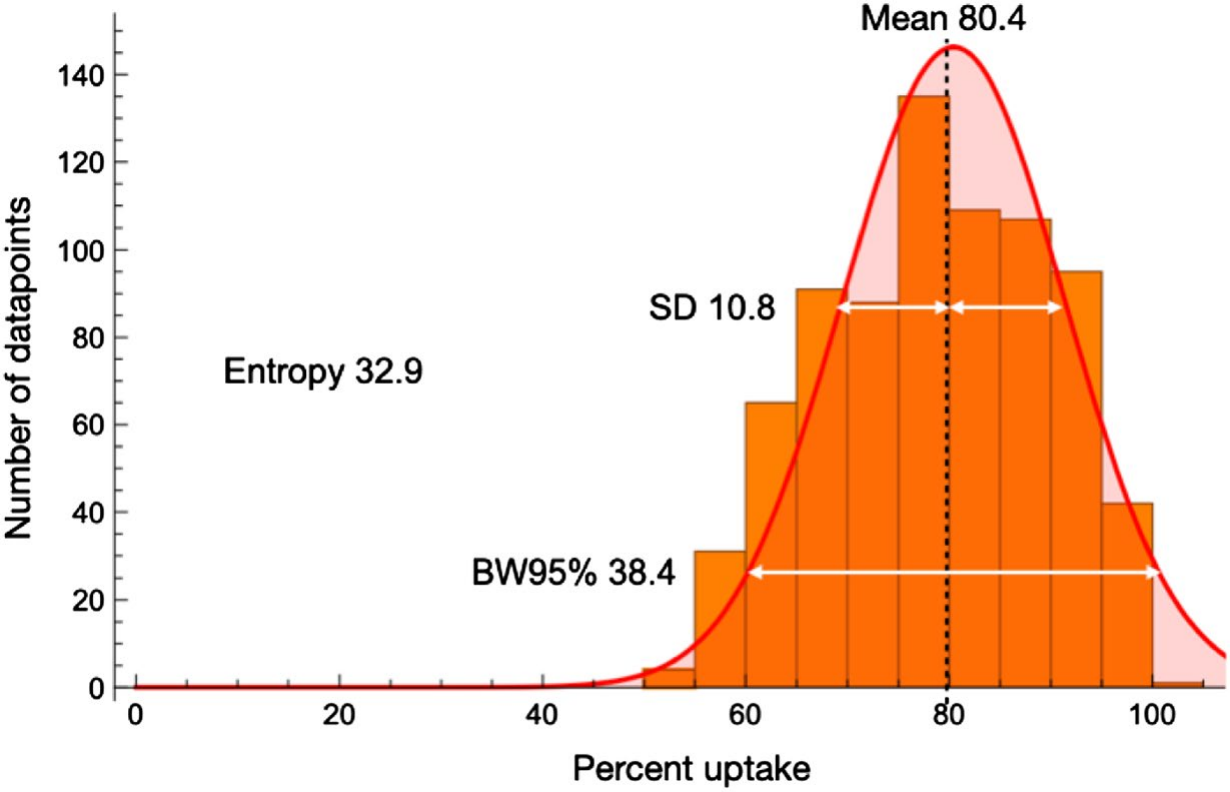
Myocardial heterogeneity in myocardial perfusion images evaluated by SD and BW95%. For each representative subject, a histogram was created to obtain the mean, standard deviation (SD), and 95% bandwidth (BW95%) of the %uptake data. The %uptake was defined as the tracer count of each pixel divided by the highest pixel value in the LV myocardium

Array maps were created by short-axis SPECT images using an algorithm of a three-dimensional segmentation model, which can export files of the datapoint array (20 rows and 48 columns) for polar map display [12]. The array maps were imported as Digital Imaging and Communication in Medicine (DICOM) format and analyzed by a program developed on Mathematica software (v. 12.3.1.0, Wolfram Research, Inc., Champaign, IL, USA). On the program, the two most apical and basal slices, which possibly included extracardiac activity and slight misalignment of slices, were excluded. The final datapoints on the array map were 768 data from 16 rows (apex to base) and 48 columns (circumferential direction). A perfusion histogram was created to observe the distribution pattern of the %uptake. Based on the histogram analysis, the SD and the BW95% were calculated (Fig. 1). The SD is defined as the regional distribution of the %uptake data, while the BW95% is defined as the bandwidth that contains 95% of the %uptake data. Entropy, an index of disorder, was defined as the summation of [fi*log(fi)]/Log(n), where f and n represent the frequency in the i-th bin and the number of bins, respectively. The entropy value ranges from 0 to 1 (0–100%), indicating complete order (0) to disorder (100) [3, 4]. All the analyses were performed automatically. Two nuclear medicine specialists classified the study subjects into two groups (non-heterogeneity group and heterogeneity group) based solely on visual assessments. They determined whether the distributions in the tomographic images and polar maps were heterogeneous or not. The finding was judged as heterogeneous if there was an uneven/patchy, mild, and minor (less than 1 segment) reduction in the accumulation of ^99m^Tc-MIBI. The discrepancy in judgment was resolved through discussion reaching a consensus.

### Statistical analyses

Continuous variables that follow a normal distribution were compared using the Student’s *t* test and one-way analysis of variance. The results were expressed as mean ± standard deviation. For continuous variables that did not follow a normal distribution, the Wilcoxon–Mann–Whitney test was used. Categorical variables were compared using either the chi-square test or Fisher’s exact test. A two-tailed *p* value of < 0.05 was considered statistically significant. Receiver operating characteristic (ROC) curve was generated, and the area under the curve (AUC) and cut-off values for each heterogeneity parameter of %uptake, such as SD, BW95, and entropy, were determined to detect the cases without visually heterogeneous distribution. The discriminative ability between the patients with cardiac disease and healthy subjects was also evaluated by ROC analysis. Inter- and intra-observer variabilities of heterogeneity parameters were analyzed using Bland–Altman analysis. We performed the analyses using JMP Pro 14.0.0 software (SAS Institute, Cary, NC, USA).

## Results

According to the visual assessments by two nuclear medicine specialists, 11 (22%) and 38 (78%) were classified into non-heterogeneity and heterogeneity groups, respectively. The clinical characteristics of the two groups are shown in Table 1. The non-heterogeneity group consisted of only healthy subjects, and all patients with cardiac disease were classified into the heterogeneity group. Interestingly, some healthy subjects were classified in the heterogeneity group. No significant differences were observed in general information and LV diameters at end-diastole in echocardiography between the two groups.

**Table 1:** Clinical characteristics of the cases in heterogeneity and non-heterogeneity groups

	Non-heterogeneity group (*n* = 11)	Heterogeneity group (*n* = 38)	*p* value
General information			
Healthy subjects, *n* (%)	11 (100)	9 (24)	< 0.01
Cardiac diseases, *n* (%)	0 (0)	29 (100)	< 0.01
Age (years)	51 (41–64)	49 (40–63)	0.39
Male, *n* (%)	4 (36)	18 (47)	0.52
Body mass index, (kg/m^2^)	22 (20–23)	20 (18–22)	0.11
Echocardiography			
LVEF (%)	66 (64–69)	58 (45–64)	< 0.01
LVDd (mm)	45 (43–47)	47 (43–53)	0.12
RI parameter			
SRS	1 (0–3)	2 (2–3)	0.05

Continuous data are expressed as median and interquartile range for nonsymmetrical variables, and crude number and percentage are used for categorical variables

*LVDd* left ventricular diameter at end-diastole, *LVEF* left ventricular ejection fraction, *RI* radio isotope, *SRS* summed rest score

**Fig. 2:**
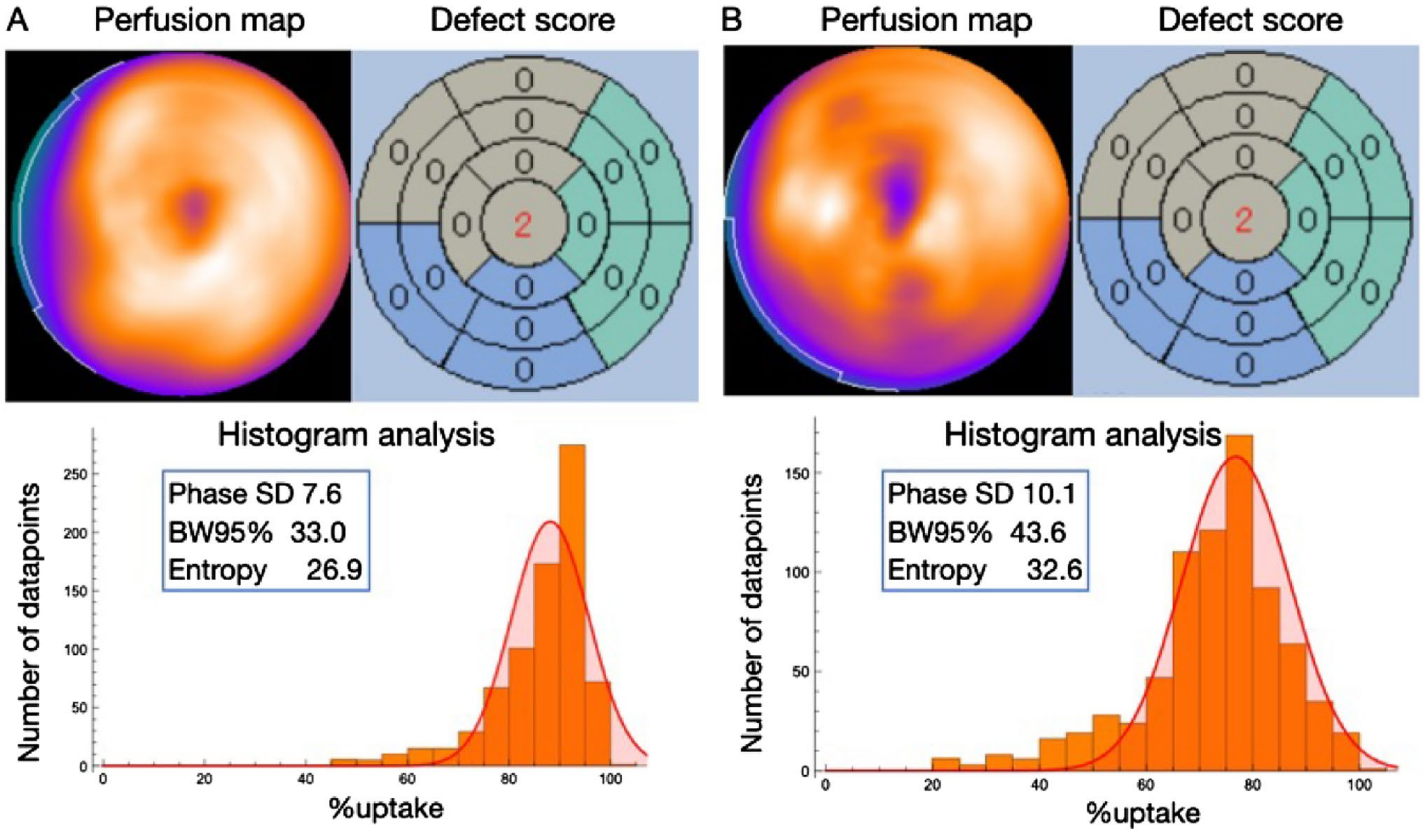
Representative cases. Panels **A** and **B** show the perfusion map, rest defect score, and histogram of myocardial perfusion SPECT in cases classified into the non-heterogeneity and heterogeneity groups, respectively. The segmental rest score (SRS = 2) remained consistent across the apical segments of both defect score maps, despite evident heterogeneity observed in Panel B

**Fig. 3:**
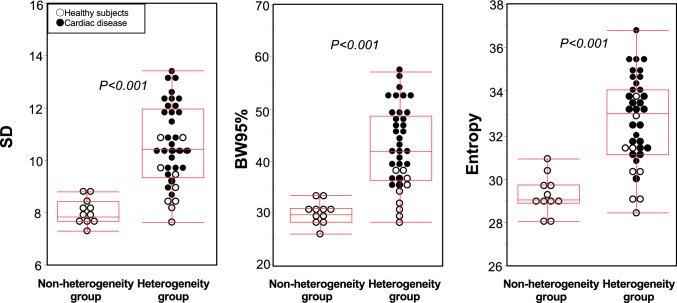
Comparisons of SD, BW95%, and entropy between the non-heterogeneity group and heterogeneity group. The cases in the heterogeneity group had significantly higher values for SD, BW95%, and entropy than those in the non-heterogeneity group (*p* < 0.05 for all). White circles represent healthy subjects, while black circles represent patients with cardiac disease

The representative cases in each non-heterogeneity and heterogeneity group are shown in Fig. 2A and B, respectively. The case in the heterogeneity group visually exhibits a heterogeneous distribution compared to the case in the non-heterogeneity group. Although the SRS in the 17-segment model of polar maps was the same in the two cases (SRS = 2), histogram analysis revealed that the histograms of % uptake were more dispersed for the heterogeneity group than for the non-heterogeneity group. Figure 3 shows SD, BW95%, and entropy of %uptake in the two groups. The SD, the BW95%, and the entropy in the heterogeneity group were significantly higher than those in the non-heterogeneity group [median values (interquartile range); 10.4 (9.3−12.0) vs. 7.8 (7.7−8.4), *p* < 0.01, 42 (36−49) vs. 30 (28−31), *p* < 0.01, and 33 (31−34) vs. 29 (29−30), *p* < 0.01, respectively]. The two groups had an overlap in values in each heterogeneity index; however, most overlapped cases were healthy subjects. In ROC curve analysis, the cut-off values for each heterogeneity index of %uptake to determine the heterogeneity group were 8.9 for SD (*p* < 0.01, sensitivity 87%, specificity 100%, AUC 0.95), 34 for BW95% (*p* < 0.01, sensitivity: 89%, specificity 100%, AUC 0.95), and 31 for entropy (*p* < 0.01, sensitivity: 82%, specificity 100%, AUC 0.94), and the AUCs of these three indices did not differ significantly. The AUCs of these three indices for discrimination between patients with cardiac disease and healthy subjects were also high (SD 0.92, BW95% 0.97, and entropy 0.92).

The coefficients of variation of inter- and intra-observer variabilities for SD, BW95%, and entropy were 7.2% and 0.5%, 9.8% and 0.8%, and 1.8% and 1.6%, respectively.

## Discussion

In this study, we found that the SD, the BW95%, and the entropy calculated by histogram analysis can quantitatively assess the heterogeneous distribution identified visually in MPI. Such quantitative assessment of the heterogeneous distribution in perfusion images has not been reported previously. As these indices differed in similarly preserved perfusion images, the assessment of heterogeneity may offer a new perspective, reflecting subtle myocardial changes in cardiac disease.

### Heterogeneity indices: SD, BW95%, and entropy

Quantifying the heterogeneity of MPI is challenging yet essential. We demonstrated that the heterogeneity of myocardial count distribution can be assessed by analyzing histograms of the SD, BW95%, and entropy. The three indices might exhibit different characteristics. The SD better captures the characteristic shape of a histogram, especially when it consists of a single peak. If the histogram displays more complex features like multiple peaks and an asymmetric distribution, BW95%, which includes the range of 2.5–97.5% of the data points, may be more appropriate. Entropy can effectively characterize the heterogeneity or homogeneity of the distribution on the map. The normal range of entropy, however, is influenced by the algorithm of the software program, particularly by data sampling method and histogram bin size [3]. In this study, all of the SD, BW95%, and entropy showed comparable AUC values when distinguishing cases with a heterogeneous distribution. The histogram distribution shares some resemblance to a normal distribution, it is likely that all heterogeneity indices of %uptake can accurately assess the heterogeneity of regional distributions observed in cardiac patients. However, when applied to patients with intermediate to large perfusion defects, the characteristics of the three indices may differ.

In terms of how reliably these indices can be reproduced, we found that both differences between different observers and within the same observer were acceptable. Notably, when a specific array plot or a polar map is given, the results for SD, BW95%, and entropy are all the same. This is because there is no manual adjustment made during the algorithm of calculating these indices. However, the variations arise due to the distribution in the polar map we created, where we did use some manual adjustment at the base of the heart. Therefore, if we were to employ a completely automatic process for the polar map, the calculated heterogeneity indices used in this study would show no differences between different observers and within the same observer.

When assessing the impact of the locations of the left ventricle, especially the inferior wall, on these heterogeneity indices, there were no specific alternations in the three heterogeneity indices in the inferior region compared to other regions. This may be because our analysis focused not on the count itself but on the variation of the counts although the inferior wall exhibited relatively low activity due to attenuation or partly due to the proximity of liver activity.

### Heterogeneity indices and patients with cardiac disease

Heterogeneity in patients with cardiac disease has been documented through various imaging modalities, including echocardiography [13], tissue Doppler ultrasound [14], cine cardiac magnetic resonance [15], magnetic resonance grid-tagged images [16], and scintigraphy studies [17]. These studies primarily depict heterogeneity in LV function or the heterogeneous distribution of myocardial perfusion by segment. They differ from our current study, where we established indices to evaluate the heterogeneity of myocardial perfusion in units of tracer counts. Heterogeneity of myocardial perfusion has been reported to be associated with heterogeneous changes in the expression of β-adrenergic receptors [18] and a nonsense mutation in the BAG5 gene [19]. Additionally, myocardial perfusion heterogeneity can be caused not only by morphological abnormalities including cardiac enlargement but also by scattered fibrosis observed in various types of cardiomyopathies, triple vessel disease with balanced ischemia, and microvascular dysfunction. The heterogeneity index proposed in this study may further improve the differentiation of these myocardial diseases or injuries.

### Visual assessments of heterogeneity

In the field of nuclear medicine, where visual assessment is often considered the gold standard, we need to include heterogeneity indices in addition to conventional variables and normal values [20]. In all indices of SD, BW95, and entropy, some overlaps between the heterogeneity group and the non-heterogeneity group were observed. Notably, most cases were healthy subjects. This may suggest that the three heterogeneity indices are better for identifying subtle heterogeneous changes that could occur even in early stages of cardiac diseases and/or diffusely involved myocardial damages.

### Limitations

This study has limitations. First, it was a single-center study, which may have reduced the power of statistical inference. Second, the cardiac patients’ cohort might be biased because we selected only patients who underwent ^99m^Tc-MIBI scintigraphy. Third, since the heterogeneous distribution is partly influenced by technological factors, we used common acquisition and processing protocols as used in clinical practice. As some preferences for reconstructing images cannot be avoided, standardization of image quality and harmonization of normal ranges may be required. However, the feasibility of quantitative indices for heterogeneity was demonstrated in this study.

## Conclusions

Heterogeneity in MPI of ^99m^Tc-MIBI can be evaluated by the SD, BW95%, and entropy using histogram analysis. These novel indices may help to identify patients with subtle myocardial changes, even in images that show preserved perfusion.
