# Correction: Novel indices representing heterogeneous distributions of myocardial perfusion imaging

**DOI:** 10.1007/s12149-024-01951-3

**Published:** 2024-06-17

**Authors:** Misato Chimura, Tomohito Ohtani, Fusako Sera, Rie Higuchi, Kenji Kajitani, Kenichi Nakajima, Yasushi Sakata

**Affiliations:** 1 Department of Cardiovascular Medicine, Osaka University Graduate School of Medicine, 2-2 Yamadaoka, Suita, 565-0871 Japan; 2 Department of Nuclear Medicine/Functional Imaging and Artificial Intelligence, Kanazawa University Graduate School of Medicine, Kanazawa, Japan

**Correction: Annals of Nuclear Medicine (2024) 38:468–474** 10.1007/s12149-024-01920-w

In Fig. 2 of this article the legend in graph should be “SD” instead of “Phase SD”; the figure should have appeared as shown in this correction.

**Fig. 2:**
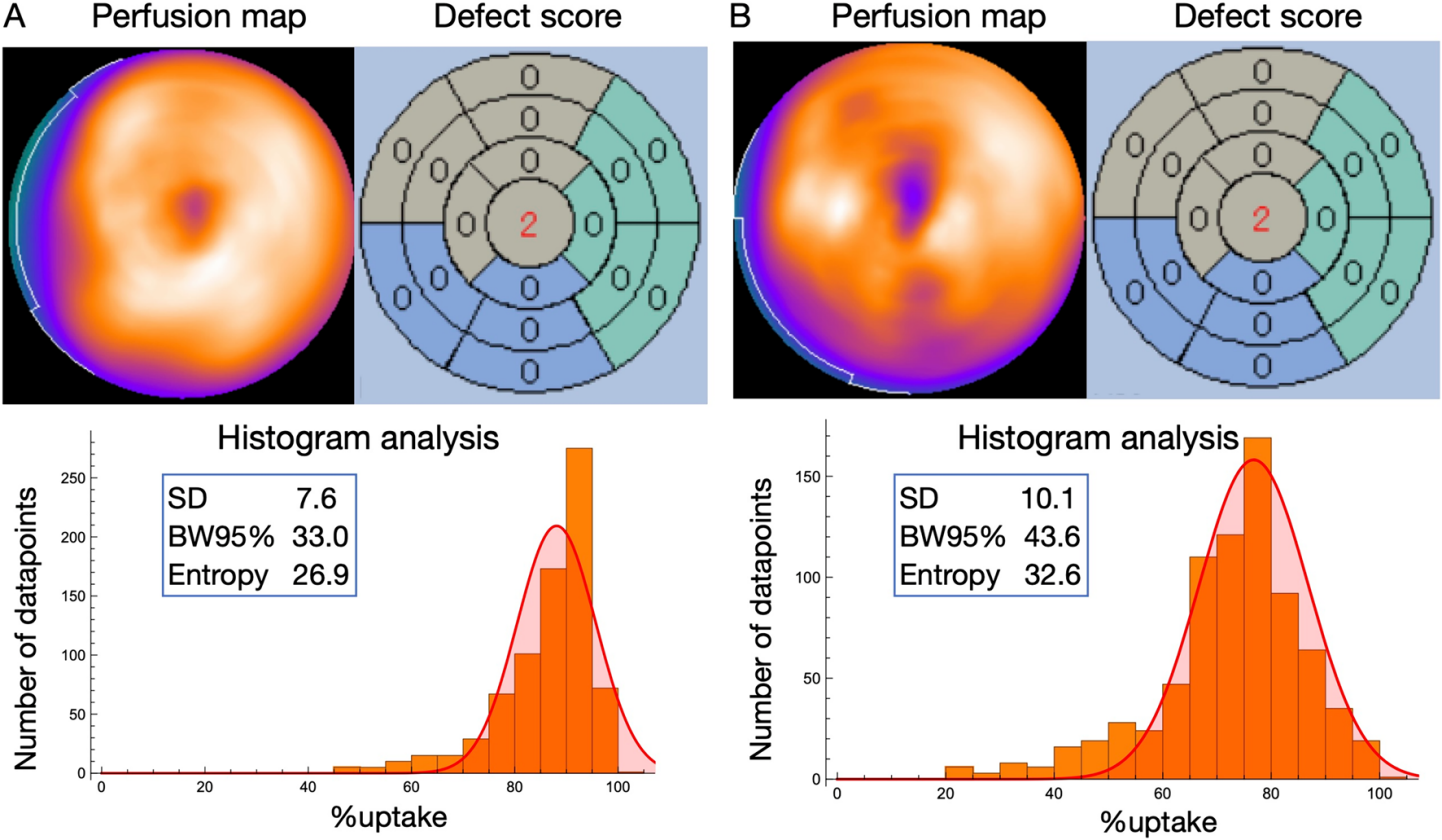
Representative cases. Panels **A** and **B** show the perfusion map, rest defect score, and histogram of myocardial perfusion SPECT in cases classified into the non-heterogeneity and heterogeneity groups, respectively. The segmental rest score (SRS = 2) remained consistent across the apical segments of both defect score maps, despite evident heterogeneity observed in Panel B

